# The Role of Recreational Physical Activity in Adherence to the Mediterranean Diet in the Greek Population: Public Health and Sustainability Implications

**DOI:** 10.3390/sports13040095

**Published:** 2025-03-25

**Authors:** Ioannis Tsartsapakis, Ioannis Trigonis, Aglaia Zafeiroudi, Olga Kouli, Vasileios Papacharisis, Dionisis Stavrousis

**Affiliations:** 1 Department of Physical Education and Sport Science, Aristotle University of Thessaloniki, 62122 Serres, Greece; dionisisstavrousis@gmail.com; 2 Department of Physical Education and Sport Science, Democritus University of Thrace, 69100 Komotini, Greece; itrigon@phyed.duth.gr (I.T.); okouli@phyed.duth.gr (O.K.); 3 Department of Physical Education and Sport Science, University of Thessaly, 42100 Trikala, Greece; 4 Division of Intercollegiate Athletics, Aristotle University of Thessaloniki, 54636 Thessaloniki, Greece; vaspap@gym.auth.gr

**Keywords:** mediterranean diet, mediterranean lifestyle, recreational physical activity, public health, climate change

## Abstract

The Mediterranean diet (MedDiet) is renowned for its health benefits and cultural significance in Mediterranean populations. The present study investigates the question of whether individuals who participate in recreational activities exhibit higher adherence to the MedDiet than those who do not engage in such activities. A cross-sectional survey was conducted with participants completing questionnaires assessing demographic characteristics, MedDiet adherence, and dietary habits. A total of 1055 participants of both sexes with an average age of 32.2 ± 10.1 years participated in the survey, and they were classified based on their engagement in recreational physical activity. Statistical analyses included independent samples *t*-tests, chi-squared tests, analysis of variance (ANOVA), and multiple regression analysis. The results demonstrated that physically active individuals exhibited significantly higher adherence to the MedDiet than non-exercisers (*p* < 0.001), supporting the hypothesis that exercise is associated with healthier dietary choices. Regression analysis further revealed that exercise, olive oil consumption, and family meals were significant predictors of MedDiet adherence. Additionally, group comparisons highlighted differences in BMI (*p* < 0.001), meal frequency (*p* < 0.001), and food preparation habits (*p* < 0.001), indicating that exercisers were more likely to adopt a holistic Mediterranean lifestyle. These findings emphasize the importance of integrating physical activity with dietary education in public health policies. Promoting an active lifestyle alongside MedDiet principles may enhance overall well-being and contribute to sustainable health strategies.

## 1. Introduction

The Mediterranean diet (MedDiet) is a dietary pattern shaped by the Mediterranean region’s cultural, historical, and environmental context [1,2]. Originating as an adaptive response to local conditions, it has gained global recognition for its health benefits [3]. This diet has significantly influenced the lifestyle patterns of Mediterranean populations throughout history [4,5].

The Mediterranean lifestyle emphasizes principles such as hospitality, harmonious coexistence, intercultural dialog, and creativity. These principles foster inclusiveness and respect for diversity through collective eating experiences during cultural events and festivals [3,6]. Characterized by a high consumption of plant foods (fruits, vegetables, bread, cereals, potatoes, beans, nuts, and seeds), minimally processed and locally grown produce, and having olive oil as the primary fat source, the MedDiet promotes a moderate intake of dairy, fish, poultry, red meat, and wine [1,7,8]. Despite ethnic and religious differences, the consumption of olive oil, seasonal vegetables, bread, and wine is a common element among Mediterranean peoples [9].

The health benefits of the MedDiet are well-documented, including reductions in cardiovascular diseases [10,11], certain cancers [12], type 2 diabetes [13], metabolic syndrome, obesity [14], cognitive decline [15], and mental health conditions [7,16]. As posited by Koliaki et al. [17], nutrition can link human health to environmental sustainability. High adherence to the MedDiet is associated with reduced environmental impacts, particularly regarding land use, soil contamination, and air pollution. Its emphasis on plant-based foods and sustainable practices makes it a potential mitigator of climate change impacts. The relationship between food production and greenhouse gas emissions, as well as the impact on climate change, is well-documented [17].

Nevertheless, the sustainability of the MedDiet is under threat from a number of factors that have a detrimental effect on the adherence of Mediterranean populations to this dietary pattern [18]. Among other factors, climate change, with its deleterious consequences, and Western lifestyle and dietary patterns appear to be two of the factors that are experiencing considerable pressure. Climate change poses significant challenges for the MedDiet’s sustainability. Rising temperatures, water scarcity, and droughts have diminished agricultural production and crop yields, affecting key components like olives, grapes, and vegetables [19]. Additionally, climate change disrupts marine ecosystems, impacting fish populations and the nutritional cohesion of Mediterranean inhabitants [20]. As demonstrated in the extant literature, climate change, in combination with prevailing socioeconomic conditions [18], is altering dietary patterns in the Mediterranean region [21,22,23]. This change in dietary patterns has the capacity to affect health, economic well-being, quality of life and the environment [24].

The environmental impact of Western diets is less well understood and has been shown to be detrimental for the environment [17]. Research indicates that there has been an increase in meat and fat consumption, and a decrease in the consumption of a MedDiet [23,25]. In competitive sports, there is a notable adoption of high-protein diets [26,27]. The modern marketing industry promotes a slim-toned body in women and a muscular, but low-fat, body in men [28,29]. A low-fat, high-protein, low-carbohydrate diet is promoted as the ideal way to achieve this [30,31]. Conversely, studies have demonstrated that the MedDiet has the capacity to enhance athletic performance, psychophysical well-being, and various physiological parameters, including weight, body composition, energy levels, and recovery capacity [3,25,32,33,34]. High adherence to the MedDiet has been linked to improved muscular endurance, strength, and cardiovascular fitness, though the effects on body composition remain inconclusive [3,32,33].

However, when the objective is health rather than appearance or weight loss, a balanced diet such as the MedDiet has been demonstrated to offer numerous benefits to the overall health of individuals compared to the Western-style diets [7,10,14,15]. Engaging in physical leisure activities has been demonstrated to increase awareness of the importance of a balanced diet [35]. Individuals who adopt an active lifestyle become more conscious of their food choices, understanding that healthy eating enhances physical performance and overall well-being [35,36,37,38]. This increased awareness often leads to increased adherence to the MedDiet. Furthermore, the integration of sustainable land cultivation with the adoption of the MedDiet has been posited as a potential contributor to climate change mitigation [36,37,38].

In consideration of the documented decline in adherence to the MedDiet and drawing upon the extant research on the salutary effects of physical activity on the MedDiet, the following hypotheses were formulated:

(A) Individuals engaged in regular exercise and recreational sports programs will demonstrate higher levels of MedDiet adherence compared to those who do not engage in physical activity.
(B) The dietary habits and lifestyle of individuals who exercise will exhibit greater alignment with the MedDiet lifestyle compared to individuals who remain physically inactive.

## 2. Materials and Methods

### 2.1. Study Design and Procedure

We conducted power analysis using the G*Power software (v. 3.1.9.4, Heinrich-Heine-Universität Düsseldorf, Düsseldorf, Germany) to determine the required sample size for our study. Based on previous research findings [39,40], we estimated the effect size at 0.02. We set the level of significance at α = 0.05. The G*Power analysis yielded the following results: critical F-value = 1.73, numerator degrees of freedom = 13, denominator degrees of freedom = 890, and a total required sample size of 904 participants.

The present study employed a cross-sectional survey design. The study was approved by the Internal Ethics Committee (IEC) of the Department of Physical Education and Sport Science at Aristotle University of Thessaloniki, Greece (ERC-019/2024), and is in line with the Declaration of Helsinki. The distribution of the questionnaires by the researchers commenced in October 2024 and concluded in January 2025. The questionnaires were primarily distributed to private and municipal gyms, recreational sports centers, and groups coordinating organized physical recreational activities. To ensure a sample of participants who had not previously engaged in recreational exercise programs, the researchers distributed the questionnaires in various locations, including work offices, discussion groups, and department stores. Prior to data collection, the researchers provided comprehensive instructions on how to complete the questionnaires, and obtained written consent from all participants. Questionnaires that were not fully completed were excluded from the study. Participants who did not provide written consent to participate were also excluded from the study. To qualify for inclusion in the study, subjects in the exercise group were required to engage in regular recreational physical exercise or physical activity for a minimum of four days per week, with each session lasting at least 45 min. Additionally, they were required to be in good physical health and not be on any medication that would prohibit them from consuming a particular food group. Examples of suitable activities include aerobics, dance, folklore dance, yoga, cross training, and calisthenics. Outdoor activities and adventure sports include orienteering, surfing, snowboarding, and caving. Racket games include tennis, paddle tennis, badminton, and squash. Participation in other activities, including but not limited to mountain biking, mountain climbing, athletics, bowling, horseback riding, skateboarding, swimming, and hiking, was permitted, as were any other forms of exercise deemed appropriate by the investigators. Participation in these programs had to have been continuous for a period of at least two years [41]. In order to be included in the non-exercise group, participants were required not to have engaged in regular exercise for a period of two years. However, they were required to be in good physical health and not be on any medication that would prohibit them from consuming a particular food group.

The investigation into adherence to the MedDiet was conducted utilizing the questionnaire developed by Panagiotakos et al. [42]. The MedDietScore ranges from 0 to 55, with low compliance defined as a score of 0 to 20, moderate compliance defined as a score of 21 to 35, and high compliance defined as a score of 36 to 55. In order to ascertain the extent to which the participants adhered to Mediterranean eating habits, a questionnaire was constructed, comprising closed-ended questions (refer to Section 2.3). In addition, a questionnaire encompassing demographic and somatometric characteristics was also compiled for the purposes of the study. Finally, participants were invited to indicate their awareness of the effects of climate change on the natural environment, as well as to specify whether or not they participated in recreational physical exercise/activity programs. According to the World Health Organization [43], physical activity is defined as any bodily movement produced by skeletal muscles that results in energy expenditure. The term encompasses all forms of movement, including those that occur during leisure time, for the purpose of transportation to and from designated locations, or as a component of professional or domestic activities [44]. Recreational physical exercise has been defined as physical activity undertaken primarily for the purpose of pleasure and improvement of health and/or renewal of the mind. Physical recreation is usually more purposeful and planned than play, but tends to have a limited organizational structure [45].

### 2.2. Participants

A total of 1055 individuals participated in the survey. Participation in the study was voluntary, and all participants provided written consent. The mean age (M) of the participants was 32.8 ± 10.1 years, and all of them were from Greece. The inclusion criteria for participation in the study were a good health status and whether or not they participated in recreational physical exercise. Following this, participants were divided into two groups: (a) an exercise group (EG) and (b) a non-exercise group (NEG). The EG consisted of a total of 568 individuals, of which 264 were males and 304 were females. The NEG comprised 487 subjects, of whom 178 were males and 309 were females. As evidenced by numerous studies, adherence to the MedDiet varies according to adult age groups [22,46]. In light of these findings, a decision was made to categorize the participants into three distinct groups (six subgroups) based on their age: EG1 and NEG1 with age from 18 to 26 years, EG2 and NEG2 with age from 27 to 40 years, and EG3 and NEG3 with age from 41 to 59 years. Participants who self-reported being professional athletes or following a restrictive dietary regime for the purpose of weight reduction were excluded from the survey.

### 2.3. Questionnaires

(A) Somatometric and demographic characteristics. The questionnaire was developed for the specific purpose of this study and comprised items such as gender, age, weight and height. The body mass index (BMI) of the participants was calculated based on their self-reported weight and height. Furthermore, the participants were asked to provide information regarding the type(s) of recreational physical activity in which they participated, the number of years of continuous exercise they had participated in, and the frequency with which they engaged in said recreational physical exercise. The questionnaire also incorporated questions regarding marital status and educational attainment. Finally, the participants were asked to respond to a closed-ended question regarding whether they had been formally informed about climate change and its effects on the planet, or whether they had attended a formal seminar on the subject.

(Β) MedDietScore. In the context of measuring adherence to the MedDiet, there is a plethora of indicators of diet quality. The present study elected to employ the scoring methodology developed by Panagiotakos et al. [42]. The MedDietScore is determined by a process of positive and negative scoring of ingredients, utilizing a composite dietary index comprising 11 components to assess adherence to the Mediterranean diet. The components encompass the primary food groups of the Mediterranean dietary regime, comprising unprocessed cereals, fruits, vegetables, potatoes, legumes, olive oil, fish, red meat, poultry, whole dairy products, and alcohol. The questionnaire utilizes an extensive scoring scale, thereby enhancing its predictive accuracy. The theoretical range of the questionnaire spans from 0 to 55. The degree of adherence to the Mediterranean diet is categorized based on the MedDietScore as follows: low compliance (0 < MedDietScore ≤ 20), moderate compliance (21 < MedDietScore ≤ 35), or high compliance (36 < MedDietScore ≤ 55). Prior studies have validated the MedDietScore for the Greek populace [47,48].

(C) Dietary habits of individuals residing in Mediterranean regions. The questionnaire was derived from the doctoral thesis of Tsartsapakis [49] and adapted for the purposes of the present study. The survey instrument consisted of nine closed-ended questions. The first question addressed the frequency of meals and snacks consumed daily. The second question sought to ascertain whether participants generally adhere to the principle of having three main meals a day. The third question posed sought to ascertain whether participants customarily partake in meals with their families, thereby underscoring the significance of traditional family bonds within the Mediterranean region. The fourth question investigated the frequency of meals consumed outside the home with friends, offering a glimpse into the social and cultural aspects of dietary habits. The fifth inquiry sought to ascertain respondents’ preferences regarding the preparation of meals at home, specifically whether they preferred to prepare their own food or relied on other individuals for sustenance consumed in the domestic environment. The sixth question focused on the consumption of organic produce and healthy superfoods, shedding light on dietary choices that promote health and sustainability. The seventh question concentrated on the ingestion of nutritional supplements (e.g., protein, creatine, amino acids). This demonstrated the necessity for increased protein intake, particularly among individuals who engage in regular recreational physical exercise. The eighth question focused on the examination of the exclusive consumption of olive oil for daily meals. Finally, the ninth question explored the main motives for some people to substitute olive oil with other oils.

### 2.4. Data Analyses

Statistical analyses were performed with the use of IBM SPSS Statistics ver. 29.0 (IBM Co., Ltd., Armonk, NY, USA). The normal distribution of the data was assessed using the Kolmogorov–Smirnov test, which indicated that the samples obtained were normally distributed. Cronbach’s alpha was used as a measure of the internal consistency of the MedDietScore (see Section 3.2.1). Continuous variables are presented as mean ± standard deviation, while categorical variables are presented as whole numbers (N) with percentages (%). Independent samples *t*-test was used to determine differences between groups for continuous variables. The chi-squared test (X^2^) was employed to analyze the contrast of categorical data. A one-way analysis of variance (ANOVA) was performed in order to test for significant differences between the six age subgroups. A Bonferroni post hoc test was used to make comparisons amongst these subgroups. Stepwise multiple regression analysis was used with the following parameters: adherence to MedDiet (MedDietScore) was considered a dependent variable, and the independent variables included exercise, gender, BMI, marital status, education and the first eight questions of the “Dietary habits of individuals residing in Mediterranean regions” questionnaire: (1) daily meals with the snacks, (2) daily main meals (no snacks), (3) eating home with the family, (4) eating with friends outside or at festivals, (5) who is cooking at home, (6) consumption of biological or superfood products, (7) consumption of proteins, creatine, amino acids and (8) exclusive consumption of olive oil. *p* < 0.05 was used as the statistical significance threshold.

## 3. Results

### 3.1. Descriptives—Somatometric and Demographic Data

As illustrated in Table 1, the somatometric characteristics of the entire sample and of participants in both the exercise group (EG) and the non-exercise group (NEG) are presented as means and standard deviations for the variables of age (years), weight (kg), height (meters), and BMI (kg/m^2^). As presented in Table 2, the means and standard deviations of the two groups (EG, NEG) and the two genders within each group for MedDiet adherence as derived from the MedDietScore scores are shown. Conversely, Table 3 presents the demographic characteristics and dietary habits of the two groups (EG, NEG), expressed as percentage frequencies (%).

In response to the question regarding the provenance of their knowledge on climate change, EG and NEG participants indicated that 8.7% (49 individuals) and 6.3% (31 individuals), respectively, had attended specialized seminars. In contrast, the percentages of the groups who cited media sources as their primary information source were significantly higher, at 91.3% (519 individuals) and 93.7% (456 individuals), respectively.

### 3.2. Parametric Analysis

#### 3.2.1. Reliability Analysis

In order to assess the internal consistency of the scales, Cronbach’s α coefficient was calculated. The MedDietScore scale demonstrated a Cronbach’s α coefficient of 0.89, which is regarded as satisfactory.

#### 3.2.2. Independent Samples *t*-Test for MedDietScore and BMI

To assess the differences in MedDietScore and BMI between the two groups, an independent samples *t*-test was conducted. Levene’s test for equality of variances was performed to check the assumption of equal variances, including MedDietScore: *F*_(1053)_ = 33.787, *p* < 0.001, BMI: *F*_(1053)_ = 278.046, *p* < 0.001. Since the Levene’s test was significant for both MedDietScore and BMI, the assumption of equal variances was violated. Consequently, the *t*-test results assuming unequal variances (Welch’s *t*-test) were reported. The results of the independent samples *t*-test indicate that there were significant differences in MedDietScore and BMI between the two groups (EG, NEG). For MedDietScore, *t*(1045.86) = 49.404, *p* < 0.001, with a mean difference of 11.588 (95% CI [11.128, 12.048]). For BMI, *t*(649.42) = −10.762, *p* < 0.001, with a mean difference of −2.189 (95% CI [−2.588, −1.789]). Specifically, the aforementioned analysis demonstrated that the EG exhibited a statistically significantly higher mean MedDiet adherence in comparison to the NEG, concurrently demonstrating a statistically significantly lower mean BMI than the NEG.

To assess the differences in MedDietScore and BMI between the men and between the women of the two groups (EG, NEG), two more independent samples *t*-test were conducted. Levene’s test for equality of variances was performed to check the assumption of equal variances (men: MedDietScore *p* = 0.001, BMI *p* = 0.001, women: MedDietScore *p* = 0.001, BMI *p* = 0.001). Since the Levene’s test was significant for both MedDietScore and BMI, for both *t*-tests, the assumption of equal variances was violated. Consequently, the *t*-test results assuming unequal variances (Welch’s *t*-test) were reported. The results indicate that there were significant differences between the men and between the women of the two groups (EG, NEG), in MedDietScore and BMI. For MedDietScore, the men of the EG exhibited a statistically significantly higher mean MedDiet adherence compared to the men of the NEG, *t*(430.778) = 30.659, *p* < 0.001, with a mean difference of 11.153 (95% CI [10.438, 11.868]). Consistent, women of the EG exhibited a statistically significantly higher mean MedDiet adherence in comparison to the women of the NEG, *t*(578.52) = 38.676, *p* < 0.001, with a mean difference of 11.933 (95% CI [11.327, 12.539]). For BMI, the men of the EG demonstrating a statistically significantly lower mean BMI than the men of the NEG, *t*(224.779) = −14.178, *p* < 0.001, with a mean difference of −3.169 (95% CI [−3.609, −2.728]). Lastly, the women of the EG exhibited a statistically significantly lower mean BMI than the women of the NEG, *t*(396.28) = −7.608, *p* < 0.001, with a mean difference of −1.980 (95% CI [−2.492, −1.468]).

#### 3.2.3. X^2^ (Chi-Squared) Analysis

In order to see the differences between the EG and NEGs for the categorical variables “education”, “marital status”, and the first eight questions of the “Dietary habits of individuals residing in Mediterranean regions” questionnaire (see Section 2.3. Questionnaires), a Pearson chi-square test was performed. The result of the Pearson chi-square test showed significant differences between the EG and NEGs for the factor marital status (X^2^ = 13. 083, df = 3, *p* = 0.004), the factor education (X^2^ = 58.753, df = 4, *p* < 0.001), and the factors daily meals with the snacks (X^2^ = 22.581, df = 2, *p* < 0.001), daily main meals (no snacks) (X^2^ = 41.127, df = 2, *p* < 0.001), eating home with the family (X^2^ = 22. 318, df = 2, *p* < 0.001), eating with friends outside or at festivals (X^2^ = 17.859, df = 2, *p* < 0.001), who is cooking at home (X^2^ = 13.607, df = 2, *p* < 0.001), consumption of proteins, creatine, amino acids (X^2^ = 34.319, df = 1, *p* < 0.001), and exclusive consumption of olive oil (X^2^ = 14.606, df = 1, *p* < 0.001). The findings of this study suggest an association between exercise and the aforementioned factors, independent of their consumption of biological or superfood products. A non-significant difference was observed between the EG and NEGs in terms of consumption of biological or superfood products (X^2^ = 0.550, df = 1, *p* = 0.459). This finding suggests that there is no association between exercise, in any form, and the consumption of biological or superfood products.

#### 3.2.4. Analysis of Variance (One-Way Anova)

In order to ascertain whether there were statistically significant differences in MedDiet adherence and BMI between the three age subgroups of the EG and NEGs, both between each group and between the two groups, a one-way analysis of variance (ANOVA) was conducted to compare the effect of age subgroups on MedDietScore and BMI. As Levene’s test was found to be significant for both MedDietScore and BMI, the assumption of equal variances was violated, indicating heteroscedasticity. Consequently, the results of the Welch ANOVA were also considered. A Bonferroni post hoc test was conducted to further examine the differences between specific groups. The results of the MedDietScore questionnaire demonstrated significant differences in adherence to the MedDiet between the six age subgroups *F*_(5,607)_ = 315.715, *p* < 0.001, η^2^ = 0.722. Post hoc comparisons using the Bonferroni test indicated that the control age subgroups (NEG1, NEG2, and NEG3) scored significantly lower on the MedDiet adherence compared to the exercise age subgroups (EG1, EG2, and EG3) (*p* < 0.001, *p* < 0.001, *p* < 0.001, respectively). No significant differences were observed among the exercise age subgroups themselves (*p* = 1.000). The analysis of variance (ANOVA) revealed significant differences in BMI scores among the groups *F*_(5,607)_ = 20.808, *p* < 0.001, η^2^ = 0.146). Post hoc comparisons using the Bonferroni test demonstrated that the BMI scores of the control age subgroups (NEG1 and NEG3) were significantly higher than those of the exercise age subgroups (EG1, EG2, and EG3). Specifically, NEG1 and NEG3 had significantly higher BMI scores compared to all exercise age subgroups (EG1: *p* < 0.001, EG2: *p* < 0.001, EG3: *p* < 0.001). Furthermore, NEG2 exhibited significantly lower BMI scores in comparison to NEG2 *(p* = 0.006) and NEG3 (*p* ≤ 0.001), yet not in relation to EG1, EG2, and EG3 (*p* > 0.50, respectively).

#### 3.2.5. Multiple Regression Analysis (Stepwise)

A stepwise multiple regression analysis was conducted for all participants, with MedDietScore as the dependent variable. The independent variables encompassed age, gender, education, marital status, BMI, exercise, daily meals with snacks, daily main meals (excluding snacks), eating at home with family, eating with friends outside or at festivals, cooking at home, consumption of biological or superfood products, consumption of proteins, creatine, amino acids, and exclusive consumption of olive oil. The final model proved to be statistically significant, with an *F*_(3,1051)_ = 1658.8, *p* < 0.001, thus explaining 82.5% of the variance in the dependent variable (*R*^2^ = 0.826, adjusted *R*^2^ = 0.825). The factors that significantly influence the MedDietScore are shown in Table 4 below:

## 4. Discussion

The present study sought to investigate the hypothesis that individuals engaging in regular recreational physical activity exhibit higher levels of adherence to the MedDiet compared to those who do not partake in exercise. Additionally, it aimed to ascertain whether these individuals maintain a lifestyle closely resembling that of Mediterranean inhabitants. Our findings demonstrated that individuals who were physically active (EG) exhibited greater adherence to the MedDiet in comparison to those who were not physically active (NEG). This robust positive relationship between physical activity and MedDiet adherence suggests a holistic adoption of health-promoting behaviors among exercisers. Physically active individuals may possess heightened health awareness and motivation to engage in behaviors that enhance overall well-being. The consistency of these findings with prior research is noteworthy. For instance, Bizzozero-Peroni et al. [33] determined that adherence to the MedDiet correlates with higher levels of physical activity, a vital health indicator throughout adulthood. Similarly, García-Hermoso et al. [35] posited that improved dietary habits, exemplified by high MedDiet adherence, are associated with enhanced physical fitness, reduced sedentary behavior, and overall better health in young adults. Additionally, research by Bonaccorsi [32] highlights the MedDiet’s role as effective nutritional support in sports and physical activity, as part of the Italian national doping prevention program. Laganà et al. [3] further explored this relationship, concluding that proper nutrition underpins good athletic performance, with the MedDiet promoting holistic development and maintaining physical fitness. The substantial disparities in BMI observed between the EG and NEGs, both among men and women, serve to further substantiate these findings. As has been thoroughly reviewed, individuals who engage in regular physical activity tend to exhibit optimal BMI levels in healthy circumstances [50,51]. In accordance with the extant literature, the EG men exhibited a BMI within the healthy range. Conversely, the NEG men, according to conventional BMI standards, demonstrated a degree of overweight. The absence of physical activity and the presence of excess weight can, in fact, give rise to numerous health and well-being concerns [52]. In contrast, the women in both the EG and NEGs exhibited significant disparities in BMI, yet their BMIs remained within the healthy range. This observation suggests that, for women, BMI is influenced by factors beyond diet and exercise [53,54].

The findings of the regression analysis showed that, in addition to exercise, factors such as exclusive consumption of olive oil and regularity of family meals were positively associated with higher adherence to the MedDiet. This suggests that physically active individuals not only adhere to dietary patterns, but also adopt lifestyle aspects of the Mediterranean way of life. The utilization of olive oil as the primary dietary fat is consistent with traditional Mediterranean dietary practices, which are abundant in monounsaturated fats beneficial for cardiovascular health [55]. The consumption of olive oil is a common characteristic found in all Mediterranean populations [56]. However, climate change has substantially impacted olive oil production over the last decade, leading to a considerable increase in price [57,58]. The present study reveals that a significant proportion of individuals in EG (33%) and NEG (44.8%) no longer exclusively consume olive oil in their diets, with the underlying factors being predominantly economic. Notably, even within the NEG, a persistent shift towards the consumption of alternative oils has been observed, with a 20.4% increase compared to 5.4% in the EG. These percentages are of particular concern as they indicate a lack of adherence to the MedDiet, evidenced by the cessation of olive oil consumption. Despite heightened awareness among the trainee participants concerning the advantages of a sustainable and healthy diet, factors such as climate change and its impact on product prices have led to a modification in their consumption patterns, resulting in diminished adherence to the MedDiet. Responses to the question on climate change indicate that only 8.7% of EG and 6.3% of NEG had participated in specialized seminars on climate change and its impact on the food chain. This finding underscores the necessity for implementing a series of specialized awareness programs aimed at enhancing public cognizance of the pernicious repercussions of climate change on their quality of life and general well-being.

Research indicates that regular family meals positively impact social relationships and are associated with healthier food choices and eating habits [59]. Our study demonstrates that the majority of EG participants (88.6%) adhere to this habit compared to 78% of NEG participants. The regression analysis shows that the daily family meal habit is a significant predictor of MedDiet adherence. The emphasis on family meals among physically active individuals underscores the cultural and social dimensions of the Mediterranean lifestyle, which have been shown to enhance mental and physical health outcomes and sustain the Mediterranean lifestyle [60]. A more in-depth examination of the cultural and social factors intrinsic to the Mediterranean lifestyle is warranted for a comprehensive understanding. The social dimension of the MedDiet, characterized by shared consumption and meal preparation, fosters an environment conducive to healthy living [61,62]. This is particularly relevant in Greek society, where family and community relations are deeply entrenched in everyday life [61].

A substantial body of research has indicated that educational attainment, in conjunction with regular physical activity, has a significant impact on the promotion of healthy dietary choices [63,64,65]. Our findings revealed a significant difference in educational levels between the EG and NEGs. Specifically, in the EG, 18% were university students, compared to 36.8% in the NEG; 57.9% were university graduates, compared to 38.6%; and 14.1% compared to 11.7% held a master’s or doctoral degree. These percentages highlight the role of education in fostering an understanding of the relationship between healthy eating, physical performance, and overall well-being. Educated individuals are more likely to comprehend the benefits of the MedDiet and integrate it into their lifestyles, reinforcing the importance of educational attainment in adopting health-promoting behaviors. This is consistent with previous studies, which have demonstrated that higher educational levels are associated with better adherence to dietary guidelines and improved health outcomes [66]. Lower educational levels and the absence of regular physical activity appear to be significant factors in the moderate adherence to the MedDiet observed in the NEG. These findings suggest that targeted educational interventions could play a crucial role in improving dietary adherence. Educational programs that emphasize the benefits of the MedDiet and physical activity could enhance awareness and motivation, particularly among individuals with lower educational attainment. Moreover, education can serve as a bridge to understanding the broader implications of diet and exercise on long-term health. For instance, by fostering critical thinking and awareness, educational initiatives can empower individuals to make informed dietary choices, understand the environmental impact of their food consumption, and recognize the importance of physical activity in maintaining health.

Finally, it is important to discuss the mean MedDietScore in the EG (M = 41.0 ± 4.26) and the fact that 48% of them reported consuming protein, amino acid, or creatine supplements. The ingestion of protein-related dietary supplements has been demonstrated to modify adherence to the MedDiet. For instance, a subject may self-report consuming meat or fish on a biweekly basis; however, the consumption of protein is often augmented through the use of supplements. The MedDiet is characterized by lower caloric content, due to its lower protein and fat content, and higher fiber and micronutrient content [67]. Consequently, individuals engaging in higher levels of physical activity may compensate for consuming larger amounts of food by consuming dietary supplements [68], potentially deviating from typical Mediterranean dietary patterns. This deviation could result in lower adherence to the Mediterranean diet, particularly if the additional dietary intake consists of non-Mediterranean foods. It can be hypothesized that this consumption of dietary supplements partially explains the EG’s MedDietScore, which, although in the range of high adherence to the MedDiet (36 < MedDietScore ≤ 55), is significantly different from the highest score.

### 4.1. Study Limitations

The present study is subject to several limitations, the details of which are outlined below. Firstly, the tool utilized for the evaluation of the Mediterranean lifestyle may demonstrate limitations with regard to validity and reliability, a circumstance which has the potential to affect the accuracy of the findings. The reliance on self-reported data for dietary habits and physical activity levels may introduce bias. Participants may overestimate or underestimate their adherence to the MedDiet or their level of physical activity. The cross-sectional design of the study limits the ability to establish causality between physical activity and adherence to the MedDiet. Longitudinal studies would be needed to confirm these relationships over time. A notable limitation of the survey was the omission of an inquiry into the participants’ income, a crucial aspect in ensuring equitable access to food. Also, in our analytical framework, we did not incorporate the analysis of the prevalence of underweight, normal weight, overweight, and obesity in addition to our estimation of the mean and standard deviation. Furthermore, the study may not have taken into account external factors that could influence both physical activity and dietary adherence, such as social support, access to recreational facilities, and the availability of MedDiet foods.

### 4.2. Implications and Applications

The study also has possible implications and applications which must be highlighted. The findings emphasize the significance of integrating physical activity with dietary guidelines in public health interventions. Consequently, the integration of physical activity and adherence to the MedDiet in public health interventions holds considerable potential for enhancing overall health outcomes. The development of educational initiatives that emphasize the benefits of the MedDiet and regular physical activity could be a fruitful avenue for targeting diverse population groups. These initiatives have the potential to raise awareness and encourage individuals to adopt healthier lifestyles. Furthermore, policymakers could utilize the study’s findings to support the formulation of policies that promote the availability and affordability of MedDiet foods, as well as access to recreational facilities, with the aim of encouraging physical activity and healthy eating. Community-based programs that emphasize the social and cultural dimensions of the Mediterranean lifestyle, such as shared meals and family activities, have the potential to enhance adherence to the MedDiet and overall well-being. The study’s findings could inform future research exploring the mechanisms underlying the relationship between physical activity and adherence to the MedDiet. Furthermore, longitudinal studies could be conducted to confirm the long-term health benefits of this integrated approach. It is recommended that healthcare professionals incorporate the study’s insights into their practice, with the aim of encouraging patients to engage in recreational physical activity and adopt the MedDiet as part of a holistic approach to health and wellness.

## 5. Conclusions

To summarize, the present study underscores the interconnection of recreational physical activity, dietary adherence, cultural and social dimensions, educational attainment, and environmental factors in promoting the Mediterranean lifestyle. Addressing these factors comprehensively can develop strategies to enhance public health and encourage sustainable, healthy living practices. By encouraging individuals to adopt a lifestyle that includes regular physical activity and a sustainable diet, we can reduce the environmental impact of our dietary choices and contribute to a healthier, more sustainable future for all.

## Figures and Tables

**Table 1:** Somatometric characteristics of the entire sample and of participants in both the exercise group (EG) and the non-exercise group (NEG).

		Age (Years)	Hight (m)	Weight (kg)	BMI (kg/m^2^)
	ν	M ± SD	M ± SD	M ± SD	M ± SD
Total Sample	1055	32.2 ± 10.1	1.74 ± 0.080	70.1 ± 13.3	23.0 ± 3.31
EG Total	568	33.8 ± 9.79	1.75 ± 0.079	67.5 ± 9.81	22.0 ± 1.85
EG men	264	35.6 ± 9.91	1.80 ± 0.058	75.7 ± 5.99	23.2 ± 1.25
EG women	304	32.2 ± 9.41	1.70 ± 0.058	60.4 ± 6.31	21.0 ± 1.62
NEG Total	487	30.3 ± 10.2	1.73 ± 0.082	73.2 ± 15.9	24.2 ± 4.15
NEG men	178	32.2 ± 11.0	1.78 ± 0.066	83.7 ± 10.7	26.4 ± 2.80
NEG women	309	29.1 ± 9.46	1.70 ± 0.078	67.1 ± 15.3	23.0 ± 4.27

EG = exercise group, NEG = non-exercise group, ν = number of participants, M = mean, SD = standard deviation.

**Table 2:** Descriptives statistics as mean and standard deviation for the MedDietScore.

Group	EG		NEG	
	ν = 568		ν = 487	
M ± SD	41.0 ± 4.26		29.4 ± 3.36	
Gender	Men	Women	Men	Women
	ν = 264	ν = 304	ν = 178	ν = 309
M ± SD	40.6 ± 4.29	41.2 ± 4.22	29.5 ± 3.34	29.3 ± 3.38

M = mean, SD = standard deviation, EG = exercise group, NEG = non-exercise group, ν = number of participants/groups.

**Table 3:** Demographic characteristics and dietary habits of the two groups (EG, NEG), expressed as percentage frequencies (%).

Group		EG		NEG	
		Frequency	Percent 100%	Frequency	Percent 100%
Gender	Men	264	46.5%	178	36.6%
	Women	304	53.5%	309	63.4%
Marital status	Single	333	59.6%	334	68.6%
	Married	217	38.2%	139	28.5%
	Divorced	18	3.2%	14	2.9%
Education	High school	57	10.0%	63	12.9%
	Un. student	102	18.0%	179	36.8%
	Graduate	329	57.9%	188	38.6%
	MSc, PhD	80	14.1%	57	11.7%
Daily meals with snacks	1 to 3/day	169	29.8%	211	43.3%
	4 to 5/day	371	65.3%	249	51.1%
	6 >/day	28	4.9%	27	5.5%
Daily main meals (no snacks)	1 meal/day	56	9.9%	114	23.4%
	2 meals/day	265	46.7%	223	45.8%
	3 meals/day	247	43.5%	150	30.8%
Eating home with the family	1 to 4 times/month	3	0.5%	2	0.4%
	2 to 4 times/week	62	10.9%	105	21.6%
	1 or more/daily	503	88.6%	380	78.0%
Eating with friends outside or at festivals	Never	59	10.3%	30	6.2%
	1 to 4 times/month	261	46.0%	184	37.8%
	1 to 3 times/week	248	43.7%	273	56.1%
Who is cooking at home	Alone	228	40.1%	246	50.5%
	Husband/wife	110	19.4%	64	13.1%
	Grandparents/other	230	40.5%	177	36.3%
Consumption of biological or superfood products	Yes	224	39.4%	203	41.7%
	No	344	60.6%	284	58.3%
Consumption of proteins, creatine, amino acids	Yes	272	47.9%	147	30.25
	No	296	52.1%	340	69.8%
Exclusive consumption of olive oil	Yes	379	66.7%	269	55.2%
	No	189	33.3%	218	44.8%
Reason for consuming other oils	Economic	158	27.9%	119	24.4%
	Way of cooking	31	5.4%	99	20.4%

EG = exercise group, NEG = non-exercise group, Un. = university.

**Table 4:** Stepwise logistic regression analysis.

Model	Un. Coefficient B	Std. Error	Beta	*t*	Sig.	95% CI Lower Upper
Constant	56.4028	2.484	-	69.590	<0.001	54.448 57.608
Exercise	10.898	0.182	0.782	59.809	<0.001	10.541 11.256
Exclusive consumption of olive oil	5.156	0.186	0.361	27.731	<0.001	4.791 5.521
Eating at home with family	0.937	0.235	0.052	3.990	<0.001	0.476 1.398

Sig. = significance level, CI = confidence interval for B, Un. = unstandardized.

## Data Availability

The data presented in this study are available upon request from the corresponding author, due to ethical and privacy reasons.

## Abbreviations

The following abbreviations are used in this manuscript:

MedDiet	Mediterranean diet
BMI	Body mass index
EG	Exercise group
NEG	Non-exercise group

## References

[1] Guasch-Ferré M., Willett W.C. (2021). The Mediterranean Diet and Health: A Comprehensive Overview. J. Intern. Med..

[2] Barros V.C., Delgado A.M. (2022). Mediterranean Diet, a Sustainable Cultural Asset. Encyclopedia.

[3] Laganà P., Coniglio M.A., Corso C., Turco V.L., Dattilo G., Delia S. (2020). Mediterranean Diet, Sport and Health. Prog. Nutr..

[4] Altomare R., Cacciabaudo F., Damiano G., Palumbo V.D., Gioviale M.C., Bellavia M., Tomasello G., Lo Monte A.I. (2013). The Mediterranean Diet: A History of Health. Iran. J. Public Health.

[5] Preedy V.R., Watson R.R. (2020). The Mediterranean Diet: An Evidence-Based Approach.

[6] Yannakoulia M., Kontogianni M., Scarmeas N. (2015). Cognitive Health and Mediterranean Diet: Just Diet or Lifestyle Pattern?. Ageing Res. Rev..

[7] Tsartsapakis I., Papadopoulos P., Stavrousis D., Dalamitros A.A., Chatzipanteli A., Chalatzoglidis G., Gerou M., Zafeiroudi A. (2024). Recreational Physical Activity and the Mediterranean Diet: Their Effects on Obesity-Related Body Image Dissatisfaction and Eating Disorders. Healthcare.

[8] Willett W., Sacks F., Trichopoulou A., Drescher G., Ferro-Luzzi A., Helsing E., Trichopoulos D. (1995). Mediterranean Diet Pyramid: A Cultural Model for Healthy Eating. Am. J. Clin. Nutr..

[9] Hachem F., Capone R., Yannakoulia M., Dernini S., Hwalla N., Kalaitzidis C. (2016). The Mediterranean Diet: A Sustainable Consumption Pattern.

[10] Bytomski J.R. (2018). Fueling for Performance. Sport. Health Multidiscip. Approach.

[11] Sanches Machado d’Almeida K., Ronchi Spillere S., Zuchinali P., Corrêa Souza G. (2018). Mediterranean Diet and Other Dietary Patterns in Primary Prevention of Heart Failure and Changes in Cardiac Function Markers: A Systematic Review. Nutrients.

[12] Kontou N., Psaltopoulou T., Panagiotakos D., Dimopoulos M.A., Linos A. (2011). The Mediterranean Diet in Cancer Prevention: A Review. J. Med. Food.

[13] Martín-Peláez S., Fito M., Castaner O. (2020). Mediterranean Diet Effects on Type 2 Diabetes Prevention, Disease Progression, and Related Mechanisms. A Review. Nutrients.

[14] Serra-Majem L., Ortiz-Andrellucchi A., Sánchez-Villegas A. (2019). Mediterranean Diet. Encyclopedia of Food Security and Sustainability.

[15] Charisis S., Ntanasi E., Yannakoulia M., Anastasiou C.A., Kosmidis M.H., Dardiotis E., Hadjigeorgiou G., Sakka P., Scarmeas N. (2021). Mediterranean Diet and Risk for Dementia and Cognitive Decline in a Mediterranean Population. J. Am. Geriatr. Soc..

[16] Sadeghi O., Keshteli A.H., Afshar H., Esmaillzadeh A., Adibi P. (2021). Adherence to Mediterranean Dietary Pattern Is Inversely Associated with Depression, Anxiety and Psychological Distress. Nutr. Neurosci..

[17] Koliaki C.C., Katsilambros N.L., Dimosthenopoulos C. (2024). The Mediterranean Diet in the Era of Climate Change: A Reference Diet for Human and Planetary Health. Climate.

[18] Biggi C., Biasini B., Ogrinc N., Strojnik L., Endrizzi I., Menghi L., Khémiri I., Mankai A., Slama F.B., Jamoussi H. (2024). Drivers and Barriers Influencing Adherence to the Mediterranean Diet: A Comparative Study across Five Countries. Nutrients.

[19] Mihailescu E., Bruno Soares M. (2020). The Influence of Climate on Agricultural Decisions for Three European Crops: A Systematic Review. Front. Sustain. Food Syst..

[20] Doney S.C., Ruckelshaus M., Emmett Duffy J., Barry J.P., Chan F., English C.A., Galindo H.M., Grebmeier J.M., Hollowed A.B., Knowlton N. (2012). Climate Change Impacts on Marine Ecosystems. Ann. Rev. Mar. Sci..

[21] Mattavelli E., Olmastroni E., Casula M., Grigore L., Pellegatta F., Baragetti A., Magni P., Catapano A.L. (2023). Adherence to Mediterranean Diet: A Population-Based Longitudinal Cohort Study. Nutrients.

[22] Mendonça N., Gregório M.J., Salvador C., Henriques A.R., Canhão H., Rodrigues A.M. (2022). Low Adherence to the Mediterranean Diet Is Associated with Poor Socioeconomic Status and Younger Age: A Cross-Sectional Analysis of the EpiDoC Cohort. Nutrients.

[23] Obeid C.A., Gubbels J.S., Jaalouk D., Kremers S.P.J., Oenema A. (2022). Adherence to the Mediterranean Diet among Adults in Mediterranean Countries: A Systematic Literature Review. Eur. J. Nutr..

[24] Clapp J. (2020). Food Security and Nutrition: Building a Global Narrative Towards 2030.

[25] Martinovic D., Tokic D., Martinovic L., Kumric M., Vilovic M., Rusic D., Vrdoljak J., Males I., Ticinovic Kurir T., Lupi-Ferandin S. (2021). Adherence to the Mediterranean Diet and Its Association with the Level of Physical Activity in Fitness Center Users: Croatian-Based Study. Nutrients.

[26] Antonio J. (2019). High-Protein Diets in Trained Individuals. Res. Sport. Med..

[27] Witard O.C., Garthe I., Phillips S.M. (2019). Dietary Protein for Training Adaptation and Body Composition Manipulation in Track and Field Athletes. Int. J. Sport Nutr. Exerc. Metab..

[28] Grogan S. (2021). Body Image.

[29] Swami V. (2015). Cultural Influences on Body Size Ideals. Eur. Psychol..

[30] Bazzano L.A., Hu T., Reynolds K., Yao L., Bunol C., Liu Y., Chen C.-S., Klag M.J., Whelton P.K., He J. (2014). Effects of Low-Carbohydrate and Low-Fat Diets: A Randomized Trial. Ann. Intern. Med..

[31] Foster G.D., Wyatt H.R., Hill J.O., Makris A.P., Rosenbaum D.L., Brill C., Stein R.I., Mohammed S., Miller B., Rader D.J. (2010). Weight and Metabolic Outcomes After 2 Years on a Low-Carbohydrate Versus Low-Fat Diet. Obstet. Gynecol. Surv..

[32] Bonaccorsi G. (2019). Mediterranean Diet as a Natural Supplemental Resource for Athletes and Physical Activity. Ann. Ig. Med. Prev. Comun..

[33] Bizzozero-Peroni B., Brazo-Sayavera J., Martínez-Vizcaíno V., Fernández-Rodríguez R., López-Gil J.F., Díaz-Goñi V., Cavero-Redondo I., Mesas A.E. (2022). High Adherence to the Mediterranean Diet Is Associated with Higher Physical Fitness in Adults: A Systematic Review and Meta-Analysis. Adv. Nutr..

[34] D’Angelo S., Cusano P. (2020). Adherence to the Mediterranean Diet in Athletes. Sport Sci..

[35] García-Hermoso A., Ezzatvar Y., López-Gil J.F., Ramírez-Vélez R., Olloquequi J., Izquierdo M. (2022). Is Adherence to the Mediterranean Diet Associated with Healthy Habits and Physical Fitness? A Systematic Review and Meta-Analysis Including 565,421 Youths. Br. J. Nutr..

[36] Medori M.C., Donato K., Stuppia L., Beccari T., Dundar M., Marks R.S., Michelini S., Borghetti E., Zuccato C., Seppilli L. (2023). Achievement of Sustainable Development Goals through the Mediterranean Diet. Eur. Rev. Med. Pharmacol. Sci..

[37] Linares C., Díaz J., Negev M., Martínez G.S., Debono R., Paz S. (2020). Impacts of Climate Change on the Public Health of the Mediterranean Basin Population—Current Situation, Projections, Preparedness and Adaptation. Environ. Res..

[38] WHO (2019). Strategy on Nutrition for the Eastern Mediterranean Region 2020–2030.

[39] Faul F., Erdfelder E., Lang A.-G., Buchner A. (2007). G* Power 3: A Flexible Statistical Power Analysis Program for the Social, Behavioral, and Biomedical Sciences. Behav. Res. Methods.

[40] Erdfelder E., Faul F., Buchner A. (1996). GPOWER: A General Power Analysis Program. Behav. Res. Methods Instrum. Comput..

[41] Thompson P.D., Arena R., Riebe D., Pescatello L.S. (2013). ACSM’s New Preparticipation Health Screening Recommendations from ACSM’s Guidelines for Exercise Testing and Prescription, Ninth Edition. Curr. Sports Med. Rep..

[42] Panagiotakos D.B., Pitsavos C., Stefanadis C. (2006). Dietary Patterns: A Mediterranean Diet Score and Its Relation to Clinical and Biological Markers of Cardiovascular Disease Risk. Nutr. Metab. Cardiovasc. Dis..

[43] WHO (2010). Global Recommendations on Physical Activity for Health.

[44] Bull F.C., Al-Ansari S.S., Biddle S., Borodulin K., Buman M.P., Cardon G., Carty C., Chaput J.-P., Chastin S., Chou R. (2020). World Health Organization 2020 Guidelines on Physical Activity and Sedentary Behaviour. Br. J. Sports Med..

[45] Hartley H. (2009). Sport, Physical Recreation and the Law.

[46] Marventano S., Godos J., Platania A., Galvano F., Mistretta A., Grosso G. (2018). Mediterranean Diet Adherence in the Mediterranean Healthy Eating, Aging and Lifestyle (MEAL) Study Cohort. Int. J. Food Sci. Nutr..

[47] Panagiotakos D.B., Pitsavos C., Arvaniti F., Stefanadis C. (2007). Adherence to the Mediterranean Food Pattern Predicts the Prevalence of Hypertension, Hypercholesterolemia, Diabetes and Obesity, among Healthy Adults; the Accuracy of the MedDietScore. Prev. Med..

[48] Panagiotakos D., Kalogeropoulos N., Pitsavos C., Roussinou G., Palliou K., Chrysohoou C., Stefanadis C. (2009). Validation of the MedDietScore via the Determination of Plasma Fatty Acids. Int. J. Food Sci. Nutr..

[49] Tsartsapakis I. (2018). The Effect of Dietary Habits, Gender, Personality Characteristics and Self-Esteem on the Body Image of Aerobic Exercisers.

[50] Jakicic J.M., Otto A.D. (2005). Physical Activity Considerations for the Treatment and Prevention of Obesity. Am. J. Clin. Nutr..

[51] Hill J.O., Wyatt H.R. (2005). Role of Physical Activity in Preventing and Treating Obesity. J. Appl. Physiol..

[52] GBD 2015 Obesity Collaborators (2018). Health Effects of Overweight and Obesity in 195 Countries over 25 Years. Yearb. Paediatr. Endocrinol..

[53] Teixeira P.J., Going S.B., Houtkooper L.B., Cussler E.C., Metcalfe L.L., Blew R.M., Sardinha L.B., Lohman T.G. (2006). Exercise Motivation, Eating, and Body Image Variables as Predictors of Weight Control. Med. Sci. Sport. Exerc..

[54] Anderson R., Anderson D., Hurst C. (2010). Modeling Factors That Influence Exercise and Dietary Change among Midlife Australian Women: Results from the Healthy Aging of Women Study. Maturitas.

[55] Covas M. (2007). Olive Oil and the Cardiovascular System. Pharmacol. Res..

[56] Willett W.C. (2006). The Mediterranean Diet: Science and Practice. Public Health Nutr..

[57] Gratsea M., Varotsos K.V., López-Nevado J., López-Feria S., Giannakopoulos C. (2022). Assessing the Long-Term Impact of Climate Change on Olive Crops and Olive Fly in Andalusia, Spain, through Climate Indices and Return Period Analysis. Clim. Serv..

[58] Kaniewski D., Marriner N., Morhange C., Khater C., Terral J.-F., Besnard G., Otto T., Luce F., Couillebault Q., Tsitsou L. (2023). Climate Change Threatens Olive Oil Production in the Levant. Nat. Plants.

[59] Fiese B.H., Schwartz M. (2008). Reclaiming the Family Table: Mealtimes and Child Health and Wellbeing. Social Policy Report. Soc. Res. Child Dev..

[60] Kushkestani M., Moghadassi M., Sidossis L. (2024). Mediterranean Lifestyle: More Than a Diet, A Way of Living (and Thriving). Endocr. Metab. Immune Disord. Drug Targets.

[61] Georgousopoulou E.N., George E.S., Mellor D.D., Panagiotakos D.B. (2020). Mediterranean Lifestyle: Linking Social Life and Behaviors, Residential Environment, and Cardiovascular Disease Prevention. The Mediterranean Diet.

[62] Medina F.-X. (2021). Looking for Commensality: On Culture, Health, Heritage, and the Mediterranean Diet. Int. J. Environ. Res. Public Health.

[63] Azizi Fard N., De Francisci Morales G., Mejova Y., Schifanella R. (2021). On the Interplay between Educational Attainment and Nutrition: A Spatially-Aware Perspective. EPJ Data Sci..

[64] Mukhamedzhanov E., Tsitsurin V., Zhakiyanova Z., Akhmetova B., Tarjibayeva S. (2023). The Effect of Nutrition Education on Nutritional Behavior, Academic and Sports Achievement and Attitudes. Int. J. Educ. Math. Sci. Technol..

[65] Spronk I., Kullen C., Burdon C., O’Connor H. (2014). Relationship between Nutrition Knowledge and Dietary Intake. Br. J. Nutr..

[66] Patino-Alonso M.C., Recio-Rodríguez J.I., Belio J.F.M., Colominas-Garrido R., Lema-Bartolomé J., Arranz A.G., Agudo-Conde C., Gomez-Marcos M.A., García-Ortiz L. (2014). Factors Associated with Adherence to the Mediterranean Diet in the Adult Population. J. Acad. Nutr. Diet..

[67] Blas A., Garrido A., Unver O., Willaarts B. (2019). A Comparison of the Mediterranean Diet and Current Food Consumption Patterns in Spain from a Nutritional and Water Perspective. Sci. Total Environ..

[68] Ruano J., Teixeira V.H. (2020). Prevalence of Dietary Supplement Use by Gym Members in Portugal and Associated Factors. J. Int. Soc. Sports Nutr..

